# Nicotine-induced brain metabolism associated with anger provocation

**DOI:** 10.1186/1744-9081-5-19

**Published:** 2009-04-24

**Authors:** Jean-G Gehricke, Steven G Potkin, Frances M Leslie, Sandra E Loughlin, Carol K Whalen, Larry D Jamner, James Mbogori, James H Fallon

**Affiliations:** 1 Department of Psychiatry and Human Behavior, University of California, Irvine, 19722 MacArthur Blvd., Irvine, California 92612, USA; 2 Department of Pharmacology, University of California, Irvine, CA, USA; 3 Department of Psychology and Social Behavior, University of California, Irvine, CA, USA; 4 Department of Anatomy and Neurobiology, University of California, Irvine, CA, USA

## Abstract

Cortico-limbic brain activity associated with anger may be susceptible to nicotine and, thus, may contribute to smoking initiation and nicotine addiction. The purpose of the study was to identify the brain regions that are most reactive to nicotine and show the greatest association with anger task performance. Twenty adult nonsmokers (9 women, 11 men) participated in two laboratory sessions to assess brain metabolism with fluoro deoxy-glucose Positron Emission Topography (FDG-PET) in response to nicotine and placebo patches during an anger provocation task. Outcome variables for the anger provocation task were reaction time, intensity and length of retaliation. Reaction time was associated with nicotine-induced changes in the left thalamus. Length of retaliation was associated with a functionally linked set of cortical and subcortical structures such as right frontal lobe, right anterior cingulate (BA 24), right uncus, left parietal lobe, left BA 11, left cingulate, left BA 25, left amygdala, left BA 30, left BA 38 and BA 9. These findings reveal the underlying brain circuitry targeted by nicotine during anger provocation.

## Findings

Cigarette smoking is a major public health problem, and angry dispositions have been identified as an important risk factor for smoking initiation and subsequent nicotine dependence [1-4]. In particular, behavioral studies revealed a smoking-anger linkage [5-8], but little is known about the underlying brain circuitry subserving such linkage.

Dysfunctional cortico-limbic brain activity associated with anger may be normalized by nicotine [4]. Functional imaging studies on regional brain activity in response to nicotine and smoking have documented the involvement of cortico-limbic structures such as the prefrontal cortex (including the dorsolateral prefrontal cortex, inferior frontal, medial frontal, and orbitofrontal gyri) [9-15], cingulate [12-16], thalamus [11,13,15,17-19], and amygdala [12,13,15,19]. Similarly, anger has been associated with activation of the medial prefrontal cortex, orbitofrontal cortex, and anterior cingulate cortex [20-22]. However, little is known about which brain areas are most responsive to the effects of nicotine during anger provocation. The elevated risk of dependence and the greater difficulty quitting may result, in part, from nicotine-associated normalization of activity of cortico-limbic circuitry [4].

The cortico-limbic circuitry is part of the "prefrontal system", which was first described by Rosvold and Schwatzbart [23] (see also review by Divac [24]) and may be particularly sensitive to nicotine modulation. The objective of the present study was to identify the brain regions within the prefrontal system that are most reactive to the effects of nicotine and show the greatest association with anger task performance in nonsmokers. Nonsmokers were studied to determine the effects of a fixed low-dose of nicotine via patch without the confounding effects of smoking and nicotine withdrawal. It was hypothesized that nicotine-induced anger task performance is associated with nicotine-induced changes in the medial prefrontal cortex, orbitofrontal cortex, and anterior cingulate cortex.

Twenty adult nonsmokers (9 women, 11 men), with a mean age of 24 (SD = 6), were recruited from the community via ads and flyers. All participants were physically and mentally healthy based on medical history obtained by a physician (JM). Similar to previous research [25], nonsmokers were defined by self-reported smoking abstinence of at least 2 years prior to participation in the study and not having smoked more than 100 cigarettes in their lifetime. All participants provided written informed consent approved by the Institutional Review Board of the University of California, Irvine.

Each participant completed two laboratory sessions in randomized order: one with a 3.5 mg low-dose transdermal nicotine patch (Nicoderm; GlaxoSmithKline) and the other with a look-alike placebo patch. A 3.5 mg nicotine patch (ie, 7 mg patch cut in half) was used to minimize potential side-effects in nonsmokers. The two laboratory sessions examined the acute effects of nicotine compared to placebo on brain metabolism using fluoro deoxy-glucose Positron Emission Topography (FDG-PET) during an anger provocation task [26]. The anger provocation task was a computerized Competitive Reaction Time task (CRT) designed to induce and measure retaliatory tendencies. Each participant competed with an ostensible "opponent" (of the same sex) to respond to a visual stimulus (ie, red square) as quickly as possible. We enhanced the saliency of the "opponent" by incorporating a video clip of this person. The participant was asked to set the level of white noise (0–105 dB) she/he wanted the opponent to receive if the opponent lost. After the participant set the noise level, a yellow square appeared as a "get ready" cue, followed by the red square. The objective was to press a mouse button as fast as possible. A scale on the left side of the monitor indicated the noise level the opponent set for the participant to receive. Provocation was manipulated by increasing the noise level presumably set by the opponent across time. There were 25 trials, with white noise presented during approximately 50% of the trials. The noise blasts were delivered through headphones for a variable length of time (set by the participant from trial to trial). Output variables were average reaction time, intensity, and length of retaliation.

Regions of interest were identified and verified with the Talairach and Tournoux atlas [27] and included areas that are associated primarily with the prefrontal system [24]. These areas cut across prefrontal, temporal and limbic regions (see Table 1).

**Table 1:** Regions of Interest

Frontal lobe
Superior frontal gyrus
Middle and medial frontal gyrus
Brodmann areas 9, 11, 24, 25, 29, 30, 32, 38, 46
Whole cingulate and anterior cingulate gyrus
Orbital gyrus
Parietal and temporal lobes
Limbic lobes
Amygdala
Hippocampus
Hypothalamus
Medial dorsal thalamic nucleus
Rectal gyrus
Subcallosal gyrus
Thalamus
Uncus
Substantia nigra-ventral tegmental area (SN-VTA)
Parahippocampus
Insula

Regions of interest were examined independently for left and right hemisphere activity

Each laboratory session lasted approximately 4.5 hours. First, the patch was worn for 3 hours. Then an intravenous injection of 5 mCi of FDG was given during the 30-minute CRT. Subsequently, the subject lay in the scanner for 1 hour (see Fallon et al. [25] for more details). Patch administration was verified by serum cotinine levels (see Table 2).

**Table 2:** Changes between nicotine versus placebo conditions in response to anger provocation

Variables	Placebo Mean (SD)	Nicotine Mean (SD)	*t*(19)	*P*≤
Cotinine Levels^a^				
Before patch	0.00 (0.00)	0.00 (0.00)	N/A	N/A
After patch	0.00 (0.00)	18.79 (24.20)	4.02	0.001
				
Region of Interest^b^				
Left BA 11	410.78 (66.19)	386.20 (68.04)	2.26	0.036
Right BA 11	406.76 (68.57)	381.19 (73.84)	2.60	0.018
Right BA 29	482.28 (63.03)	503.19 (65.90)	2.31	0.032
Right Rectal Gyrus	424.02 (65.31)	388.98 (92.56)	2.46	0.024
				
CRT				
Reaction time^c^	284.57 (70.78)	281.52 (77.02)	0.17	NS
Intensity of Retaliation^b^	5.53 (2.03)	5.59 (2.16)	0.13	NS
Length of Retaliation^c^	290.50 (213. 91)	319.92 (241.93)	0.82	NS

^a ^in ng/ml

^b ^in arbitrary units

^c ^in ms

For the PET data, normalized metabolic averages per anatomical region per subject were used. The metabolic averages were rescaled in order to reduce inter-individual variability in brain metabolism, and the resulting absolute metabolic averages per anatomical region per subject were used. Rescaling was achieved by taking the mean of all the brain regions within a subject (ie, global mean) and scaling it to the canonical normal global cerebral blood flow (50 ml/min/dl). This is a common technique used when analyzing PET data and provides some protection against false positives from global cerebral blood flow changes under a treatment condition [25,28]. Preliminary analyses using *t*-tests revealed that BA 11 and right rectal gyrus metabolism was significantly reduced under nicotine compared to placebo patches, whereas BA 29 showed the opposite pattern (see Table 2). No other significant differences were found [see Additional file 1].

For the anger provocation task, the CRT outcome measures of reaction time, intensity and length of retaliation were averaged across the duration of the task. No significant mean differences were found in anger task performance between nicotine and placebo conditions, perhaps due to the low-dose nicotine patch (see Table 2). Group means, however, do not reflect individual differences in brain metabolism in response to nicotine. Therefore, stepwise linear regression models were used to examine delta scores for each outcome variable in order to contrast nicotine with placebo conditions (nicotine – placebo patch). Stepwise linear regression models (SPSS 13.0) examined which areas of the prefrontal system under nicotine compared to placebo predicted CRT outcome variables in response to nicotine versus placebo. Dependent variables were the total scores on CRT outcome variables. Predictor variables were the regions of interest. The inclusion criterion used in each regression model was a probability value of 0.05. The exclusion criterion was a probability value of 0.06. To adjust for the number of brain regions, alpha was Bonferroni-corrected at 0.0008.

The results from the regression models revealed that participants who showed nicotine-induced changes in anger task performance also showed changes in brain metabolism. More specifically, nicotine-induced improvements in reaction time were associated with decreased brain metabolism in response to nicotine in the left thalamus. This area of the brain explained 56% of variance for reaction time (R = 0.75, R^2 ^= 0.56, F change (1, 16) = 20.59, p < 0.0001).

Nicotine-induced reductions in length of retaliation were associated with increased brain metabolism in response to nicotine in the left cingulate, left amygdala, right frontal lobe, left BA 30, and left BA 38. In particular, the amygdala has been shown to modulate prefrontal regional activity [29]. However, our subjects included both women and men so that some underlying amygdaloid contribution may have been diluted by mixed lateralization components in women and men [30,31]. Positive associations were found between nicotine-induced reductions in length of retaliation and nicotine-induced reduction in brain metabolism in the orbital-frontal areas (ie, right uncus, left BA 9, right anterior cingulate (BA 24), left BA 25, right BA 9, and left BA 11) and left parietal lobe. These predictors explained 100% of the variability in length of retaliation (R = 1.00, R^2 ^= 1.00, F change (1, 5) = 66.69, p < 0.0001). No significant associations were found for intensity of retaliation. See Table 3 for more details.

**Table 3:** Stepwise linear regression for reaction time and length of retaliation

Dependent Variable	Predictors^a^	*Beta*	*t*	*P****<***	*R*^2^	*Collinearity*^b^
Reaction Time	Left Thalamus	-0.750	4.538	0.0001	0.563	1.000
						
Length of Retaliation						
	Left Cingulate Gyrus	-0.599	39.660	0.0001	1.000	1.218
	Right Uncus	1.068	107.318	0.0001		1.649
	Left Amygdala	-0.349	43.520	0.0001		1.906
	Left BA 9	0.679	56.707	0.0001		2.241
	Right Frontal Lobe	-0.635	45.924	0.0001		2.950
	Right BA 24	0.245	19.426	0.0001		3.281
	Left BA 30	-0.319	27.823	0.0001		3.479
	Left Parietal Lobe	0.471	34.911	0.0001		4.269
	Left BA 25	0.330	19.769	0.0001		5.069
	Right BA 9	0.238	16.405	0.0001		5.805
	Left BA 11	0.190	10.866	0.0001		9.191
	Left BA 38	-0.099	8.167	0.0001		10.802

^a ^The order of the predictors reflects the order of the stepwise linear regression

^b ^Condition Index (i.e., values above 15 may indicate possible collinearity problems)

**Figure 1:**
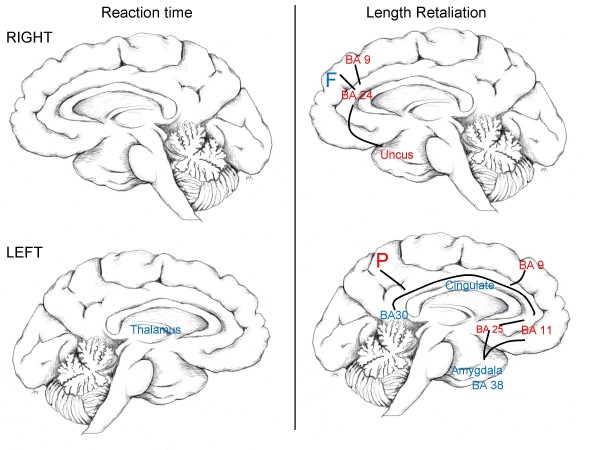
**Mid-sagittal drawing of cortical-limbic brain circuitry associated with anger provocation**. Blue areas indicate decreased nicotine-induced brain metabolism associated with increased nicotine-induced task performance. Red areas indicate increased nicotine-induced brain metabolism associated with increased nicotine-induced task performance. Lines represent implied circuitry based on well-documented anatomical connectivity. F = frontal lobe; P = parietal lobe

The involvement of generalized cortical and subcortical areas may imply that nicotine-induced changes in anger task performance correlate with nicotine-induced changes in higher cortical and subcortical functioning responsible for orienting, planning, and processing of emotional stimuli. Our findings identify a circuitry (see Figure 1), that is primarily involved in the processing of emotional stimuli [29,32-41] and complex adaptive, motivated behaviors, including the regulation of emotion [20,22,42-46].

The present findings on nicotine-induced brain metabolism in nonsmokers emphasize the role of negative affect, particularly anger, in susceptibility to nicotine. Moreover, the findings suggest that nicotine may critically regulate brain areas that are involved in the inhibition of negative emotions such as anger [43]. Deficits in anger management may be a risk factor for smoking initiation and nicotine addiction, which may highlight the importance of improving affect regulation as a potential smoking prevention and cessation strategy [4]. Novel neurotherapeutic and behavioral treatments (e.g., anger management training) that affect the cortical and limbic brain areas may aid smoking cessation efforts in anger provoking situations that increase withdrawal and tobacco cravings.

## Competing interests

The authors declare that they have no competing interests.

## Authors' contributions

JG conceptualized and coordinated the study, conducted the statistical analysis, and drafted the manuscript. SP directed the PET scanning and data processing. FL and SL initiated the research study, contributed to the research design, and reviewed drafts. CW and LJ aided in the conceptualization of the study, the interpretation of the data, and reviewed drafts. JM aided in the study coordination, obtained medical histories from participants, and reviewed drafts. JF identified and interpreted the ROIs with regards to task performance and aided in drafting Figure 1.

## Supplementary Material

Additional file 1 **Metabolic averages in response to nicotine versus placebo associated with anger provocation**. The data provide means and standard deviations for each region of interest in response to nicotine and placebo. Click here for file
