# Supplementary material for: Nicotine-induced brain metabolism associated with anger provocation
Source: Behav Brain Funct. 2009 Apr 24;5:19. doi: 10.1186/1744-9081-5-19 (PMC2680866; doi:10.1186/1744-9081-5-19)
Supplement: Additional file 1 — Metabolic averages in response to nicotine versus placebo associated with anger provocation. The data provide means and standard deviations for each region of interest in response to nicotine and placebo. [file 1744-9081-5-19-S1.doc]

**Table**  Metabolic averages in response to nicotine versus placebo associated with anger provocation

**Region of Interest Placebo Nicotine *t* (19) *P***

Mean (SD) Mean (SD)

Left Amygdala 299.87 (41.07) 297.60 (37.56) 0.26 0.796

Left Anterior Cingulate 356.67 (65.20) 360.19 (65.03) 0.48 0.634

Left BA 11 410.78 (66.19) 386.20 (68.04) 2.26 0.036*

Left BA 24 388.16 (86.63) 406.46 (85.85) 1.79 0.089

Left BA 25 398.09 (46.90) 389.30 (49.87) 1.14 0.268

Left BA 29 507.35 (35.34) 523.85 (47.55) 1.95 0.067

Left BA 30 481.90 (40.21) 495.69 (53.20) 1.56 0.137

Left BA 32 458.79 (62.68) 466.85 (61.67) 1.09 0.303

Left BA 38 305.52 (43.79) 294.82 (39.66) 1.64 0.118

Left BA 46 239.89 (117.86) 218.91 (116.22) 1.36 0.190

Left BA 9 463.11 (58.34) 458.27 (59.51) 0.67 0.513

Left Cingulate 378.56 (52.33) 384.21 (53.17) 0.73 0.475

Left Frontal Lobe 393.28 (83.58) 393.62 (85.24) 0.79 0.938

Left Hippocampus 261.90 (40.69) 259.16 (48.22) 0.27 0.789

Left Hypothalamus 271.46 (44.04) 252.85 (56.15) 1.32 0.203

Left Insula 510.18 (30.67) 521.00 (36.52) 1.29 0.213

Left Limbic Lobe 358.50 (31.32) 361.63 (33.59) 0.64 0.528

Left Middle Frontal Gyrus 451.83 (71.67) 445.89 (75.74) 0.55 0.588

Left Medial Dorsal Nucleus 553.19 (59.57) 558.12 (62.72) 0.36 0.726

Left Medial Frontal Gyrus 483.95 (37.09) 492.73 (38.67) 1.66 0.113

Left Orbital Gyrus 360.89 (99.92) 348.88 (81.91) 0.72 0.482

Left Parietal Lobe 461.80 (39.19) 466.04 (48.80) 0.49 0.628

Left Parahippocampal Gyrus 297.30 (23.40) 298.40 (31.25) 0.18 0.861

Left Rectal Gyrus 426.37 (63.18) 400.60 (82.09) 1.77 0.092

Left Subcallosal Gyrus 411.84 (53.57) 413.06 (53.16) 0.20 0.840

Left Superior Frontal Gyrus 479.78 (50.17) 474.08 (51.39) 0.77 0.448

Left Temporal Lobe 404.43 (19.03) 405.24 (26.09) 0.16 0.876

Left Thalamus 417.67 (35.04) 425.20 (36.80) 1.51 0.148

Left Uncus 300.57 (34.80) 292.84 (30.61) 1.46 0.160

Left SN-VTA 369.72 (58315) 373.57 (73.28) 0.29 0.775

Right Amygdala 276.30 (48.09) 288.57 (58.82) 1.19 0.250

Right Anterior Cingulate 409.85 (63.01) 412.30 (63.52) 0.28 0.780

Right BA 11 406.76 (68.57) 381.19 (73.84) 2.60 0.018*

Right BA 24 420.00 (80.54) 431.46 (85.36) 1.20 0.246

Right BA 25 265.81 (44.74) 262.76 (47.33) 0.52 0.611

Right BA 29 482.28 (63.03) 503.19 (65.90) 2.31 0.032*

Right BA 30 536.58 (45.70) 553.28 (50.20) 2.45 0.024

Right BA 32 510.94 (59.31) 517.01 (59.23) 0.68 0.507

Right BA 38 314.62 (33.56) 311.00 (39.02) 0.55 0.592

Right BA 46 362.03 (152.85) 344.29 (157.29) 0.93 0.364

Right BA 9 508.98 (63.93) 507.46 (70.01) 0.20 0.841

Right Cingulate 402.02 (42.93) 409.17 (46.58) 1.14 0.269

Right Frontal Lobe 434.54 (34.07) 445.02 (36.15) 1.92 0.070

Right Hippocampus 237.01 (42.49) 246.96 (33.70) 1.18 0.251

Right Hypothalamus 261.38 (56.08) 255.71 (54.38) 0.58 0.567

Right Insula 484.79 (47.34) 497.67 (48.20) 1.64 0.118

Right Limbic Lobe 383.26 (30.03) 389.14 (27.13) 1.39 0.179

Right Middle Frontal Gyrus 488.86 (55.33) 490.23 (63.68) 0.17 0.866

Right Medial Dorsal Nucleus 523.15 (63.92) 528.97 (52.81) 0.54 0.598

Right Medial Frontal Gyrus 508.01 (25.71) 517.20 (30.81) 1.64 0.118

Right Orbital Gyrus 370.00 (108.35) 349.76 (110.60) 1.17 0.257

Right Parietal Lobe 490.78 (40.92) 494.61 (41.68) 0.59 0.566

Right Parahippocampal Gyrus 292.42 (28.54) 296.83 (26.03) 0.93 0.366

Right Rectal Gyrus 424.02 (65.31) 388.98 (92.56) 2.46 0.024*

Right Subcallosal Gyrus 399.24 (62.56) 399.13 (56.41) 0.02 0.986

Right Superior Frontal Gyrus 485.20 (49.96) 480.60 (50.76) 0.65 0.520

Right Temporal Lobe 404.87 (16.96) 409.84 (20.74) 1.26 0.222

Right Thalamus 418.33 (36.91) 427.67 (30.33) 1.76 0.096

Right Uncus 283.25 (36.03) 274.27 (33.68) 1.95 0.066

Right SN-VTA 424.98 (68.36) 420.21 (56.42) 0.32 0.749
